# Combinatorial CRISPR/Cas12a excises targeted loci in the mammalian genome at very low efficiency

**DOI:** 10.64898/2026.09.21.753273

**Published:** 2026-09-22

**Authors:** Nazanin Esmaeili Anvar, Zsofia M Szegletes, Ryan J Steger, Abby V McGee, Sidney Wang, John G Doench, Traver Hart

## Abstract

The CRISPR/Cas12a system has emerged as a highly useful tool for genomic editing due to its ability to process multiple guide RNAs (gRNAs) from a single promoter. In this study, we explored the potential of combinatorial CRISPR/Cas12a to excise targeted loci in the mammalian genome by inducing double-strand breaks (DSBs) upstream and downstream of targeted exons. We designed a library of gRNA pairs targeting introns flanking 54 exons of 18 essential genes and 17 exons of 10 nonessential genes, aiming to assess the efficiency of Cas12 in excising targeted loci. While single gRNAs directly targeting essential exons produced expected fitness defects in cells, pairs of guides targeting flanking intronic regions resulted in only trivial viability effects, suggesting that excision rarely occurred. Further analysis showed no meaningful correlation between gRNA efficiency, orientation, GC content, or cut site geometry and the success of locus excision. These findings indicate that, under the conditions tested, the Cas12a system does not yield locus excision at the efficiency required for sensitive negative selection assays.

## Full Text Availability

The license terms selected by the author(s) for this preprint version do not permit archiving in PMC. The full text is available from the preprint server.

